# Age-related increase in muscle stiffness is muscle length dependent and associated with muscle force in senior females

**DOI:** 10.1186/s12891-021-04519-8

**Published:** 2021-09-27

**Authors:** Jingfei Xu, Siu Ngor Fu, François Hug

**Affiliations:** 1 Department of Rehabilitation Medicine, West China Hospital, Sichuan University, Chengdu, PR China; 2 Department of Rehabilitation Sciences, the Hong Kong Polytechnic University, Yuk Choi Road, Kowloon, Hong Kong China; 3 Institute for Disaster Management and Reconstruction, Sichuan University, Chengdu, PR China; 4 University of Nantes, Faculty of Sport Sciences, Laboratory “Movement, Interactions, Performance” (EA 4334), Nantes, France; 5 InstitutUniversitaire de France (IUF), Paris, France

**Keywords:** Muscle tension, Elastography, Ultrasound, Quadriceps femoris, Peak torque

## Abstract

**Background:**

In aging, muscle stiffness is considered as one of the factors associated with the reduction of force generation capability. There have been inconsistent findings on age-related alteration in the passive stiffness of quadriceps muscle in the female adults. Thus, the aim of this study was to determine the effect of aging on the shear moduli of the superficial muscle heads of the quadriceps and to explore its relationship with knee extension force.

**Methods:**

Passive shear moduli of the rectus femoris (RF), vastus lateralis (VL), and vastus medialis (VM) were measured at rest using shear wave elastography in 20 young and 20 senior female adults. Measurements were repeated at four knee joint positions, that is, 30°, 60°, 90°, and 105° of knee flexion. Maximal isometric voluntary knee extension force was assessed at 30°, 60°, and 90° of knee flexion.

**Results:**

As per our findings, senior adults were determined to have significantly higher passive muscle shear moduli in the RF (by 34% – 68%; all *p* < 0.05) and the VL muscle heads (by 13%–16%, all *p* < 0.05) at and beyond 60° of knee flexion. Age-related increase in the VM was evident at 105° knee flexion (by11%, *p* = 0.020). The RF shear modulus was negatively correlated to the maximal isometric voluntary contraction force measured at 60° (*r* =  − 0.485, *p* = 0.030) in senior adults.

**Conclusions:**

Senior female adults had greater passive stiffness at the superficial muscle heads of the quadriceps muscles when measured at long muscle length. Among the senior female adults, the passive stiffness of RF has been determined to have a negative association with the knee extensor force only at 60° knee flexion. No significant association was noted for other angles and muscles.

## Background

In aging, a decrease in muscle force has been assumed to be mainly induced by muscle atrophy [1, 2]. However, muscle atrophy cannot completely account for the age-induced force decrease as the force decrease (40%) has been noted to be often larger than the atrophy sign (23%) [3, 4]. The change of microstructure in a single muscle fiber, such as decreased myosin protein, slowing of myosin-actin cross-bridge in single fiber [5], reduced cross-sectional area of myosin heavy chain II fibers, and decrease in intermyofibrillar mitochondrial size with aging [6], might have contributed to the age-related muscle force decrease. In addition, muscle properties like passive muscle stiffness could also influence muscle force [7, 8] as well as muscle performance in the elderly [9]. A numerical study has demonstrated a causal association between the age-related increase in passive muscle stiffness and the reduced force generation capability with aging [10].

However, the finding that age is related to alteration of muscle stiffness is not always a consistent result. Previous animal studies have revealed higher stiffness of the aged soleus muscle [11, 12]. Similarly, the single fiber of isolated vastus lateralis (VL) muscle from an older person was noted to have greater passive stiffness compared to a younger one [13, 14]. Using qualitative ultrasound elastography in vivo, a greater muscle stiffness in biceps brachialis muscle, rectus femoris (RF), and gastrocnemius muscles was observed in elderly people [15, 16]. However, contrary results have been reported as well. For example, lower muscle stiffness was observed in the four heads of quadriceps muscle [17, 18], and comparable level of stiffness in several leg muscles has been reported in aged participants [19–22]. According to a previous study [16], age-related modulation in muscle stiffness was often influenced by muscle length, which was dependent on joint angles in vivo. It further revealed that aged muscle was stiffer than young muscle at long muscle length. However, how the joint position affects muscle stiffness was not considered in most reported studies. When the muscle length was taken into account, the muscle stiffness of triceps surae muscle in old participants was equal to that of young participants at 0° and 15° ankle dorsiflexion [23] and less than the young ones at maximal dorsiflexion [24]. Therefore, the length-dependent modulation of age on muscle stiffness has not been clarified as a whole muscle in vivo.

When the stiffness of quadriceps femoris muscle was measured at short muscle length, its relationship with extension knee force was not observed [15]. Similar result was found in medial gastrocnemius as well [25]. It should be noted that muscle stiffness was measured only at a given muscle length in these studies. As muscle stiffness was length dependent [26], it is thus necessary to explore the association between muscle stiffness and muscle force generation capacity at different muscle lengths.

In this study, our primary aim was to determine the effect of aging on the shear moduli of the superficial muscle heads of the quadriceps. Second aim was to explore the relationship between muscle shear moduli of the superficial heads of the quadriceps and knee extension force in the senior female adults. The hypotheses were senior female adults would present with greater passive muscle stiffness at long muscle length, and passive muscle passive stiffness would be negatively associated with muscle force.

## Methods

In total, 20 senior healthy females (age range: 50–70 years) and 20 young healthy females (age range: 20–32 years) were included in this study. The participants’ demographic information is summarized in Table 1. All participants were free of knee pain or injury and have no history of knee surgery. They did not engage in any regular exercise training or high-intensity sport activities.

**Table 1:** The demographic information of participants (means ± SD)

Variables	Young	Senior	*p*
Number	20	20	
Age (y)	25.4 ± 2.8	58.4 ± 5.5	< 0.001
Body weight (kg)	54.3 ± 4.1	53.6 ± 4.6	0.600
Height (cm)	161.4 ± 3.7	155.1 ± 4.8	< 0.001

### Passive muscle shear modulus measurements

**Fig. 1:**
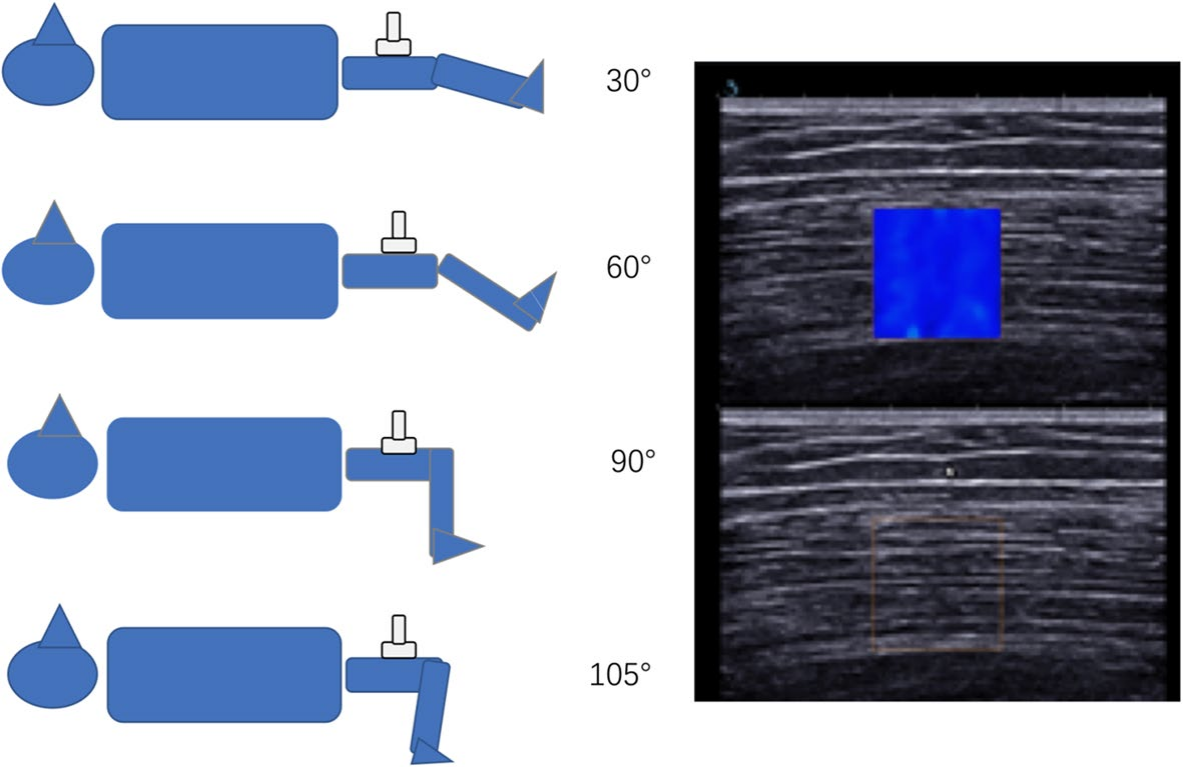
The participant position during measurements. A typical image of muscle shear modulus of vastus medialis (VM) muscle is presented

The passive muscle stiffness was first measured. The participants were positioned supine on the plinth of the isokinetic dynamometer (Cybex, Medway, MA, USA) with hip at 0° and neutral rotation, as the pelvis, trunk, and thigh were stabilized with straps. The knee joint rotation center (lateral femoris condyle) was aligned with the rotation center of the isokinetic dynamometer. Measurements were taken with the knee joint positioned at 30º, 60º, 90º, and 105º of knee flexion (0° being the knee extended) in sequence in order to avoid the stretching effect from muscle length (Fig. 1). The knee was then passively positioned at each angle and remained for 30 s before measurement started. Participants were instructed to remain relax during the whole procedure.

The muscle shear modulus (an index for muscle stiffness) of the dominant leg was assessed using an ultrasound scanner coupled with a 4–15 MHz linear transducer (AixplorerV4.0; Supersonic Imagine, Aix-en-Provence, France). The elastography machine was then set to the musculoskeletal preset. A well-trained examiner in musculoskeletal ultrasound examination performed all the measurements. The following three superficial heads of the quadriceps femoris muscle were then measured: RF, VL, and vastus medialis (VM). The measurement locations were marked on the skin with a waterproof pen before the test. For VM and VL, the transducer was placed 1/5 and 1/3 of the distance from the midpoint of medial and lateral patella border to the anterior superior iliac spine, respectively. For RF, the transducer was placed 1/2 of the distance from anterior superior iliac spine to the midpoint of the superior tip of the patella [4, 27]. The transducer was held perpendicularly to the skin with plentiful gel, and slight pressure was exerted on the skin. The transverse images were obtained to confirm the right muscle was measured; then, the longitudinal images were captured at one sample per second which was the temporal resolution of the current shear wave elastography version, and the spatial resolution was 1 × 1 mm. The probe was aligned with either the muscle fiber direction (VL and VM) or the muscle shortening direction (RF). Five continuous elastography maps were acquired in 5 s. During each measurement, the square color box was adjusted accordingly depending on the muscle thickness.

### Maximal isometric voluntary contraction (MIVC) test

After elastography measurements, MIVC of knee extension assessment was performed. The position of participants was the same as that of the passive stiffness measurement. Prior to measurement, the participants were instructed to perform two submaximal contractions for warm-up and to be familiar with the test procedure. Each participant conducted three 5-s maximal isometric voluntary contractions, with a 2-min interval between each contraction. The MIVC was assessed at three knee angles (30°, 60°, and 90° knee flexion; 0° = full extension). Due to the discomfort of the joint, the MIVC at 105° was not measured. The maximum peak torque of the three contractions captured at each angle was considered as MIVC torque at this angle.

### Data reduction and analysis

The shear wave elastography data were exported in mp4 format and sequenced in png. Image processing was performed using a custom Matlab script (MathWorks, Natick, MA). Each image was then carefully inspected for artifacts. If artifacts were present in any image, the region of interest was reduced in size to remove the artifact. The colored 2D maps were converted into shear modulus values, and the shear modulus values were averaged over the map and over the five images for further analysis.

Statistical analysis was conducted using SPSS 21.0 software package (New York, USA). Independent sample t-test was performed to compare the difference of basic demographic information and MIVC between young and senior adults. The MIVC comparison between the two age groups was analyzed via a two-way repeated measures ANOVA with knee angle as within-subject factors and group (young and senior adults) as between-subject factors. A three-way repeated measures ANOVA with muscle heads and knee angle as within-subject factors and group (young and senior adults) as between-subject factors was used to compare the shear moduli of the two groups. Post hoc analyses were performed when appropriate using the Bonferroni method. The correlations between shear modulus and the MIVC of quadriceps femoris muscle were calculated using Pearson’s coefficient if the data were normally distributed; otherwise, Spearman correlation coefficients were calculated. Statistical significance level of *p*-value was set to be less than 0.05.

## Results

**Fig. 2:**
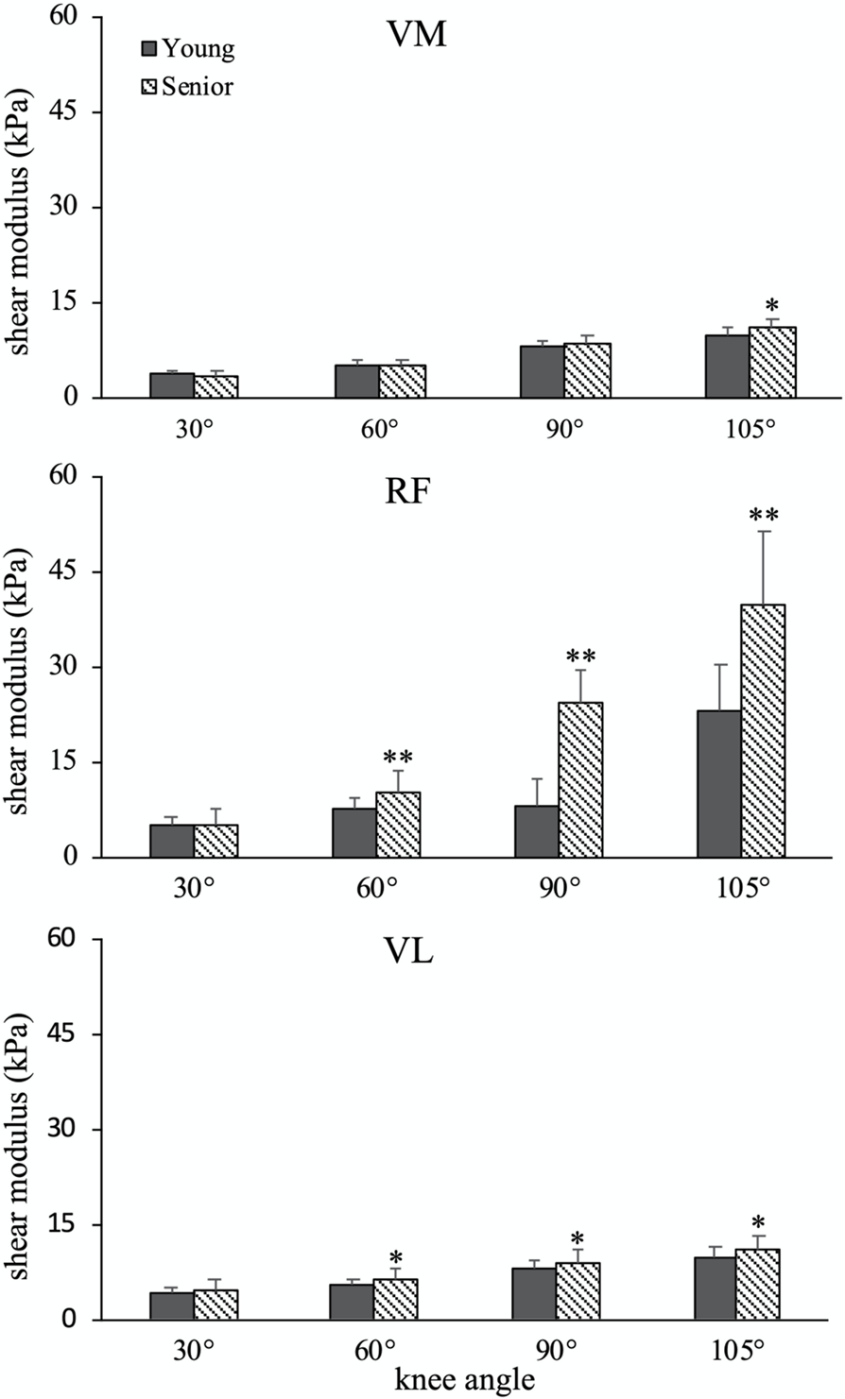
The shear moduli of VM, RF, and VL of the young and senior adults at different knee angles. Compared to young adults, the senior adults exhibited significantly greater shear moduli when assessed over 60° knee flexion for the RF and VL muscles and at 105° of knee flexion for the VM. *: *p* < 0.05, **: *p* < 0.01. VM: vastus medialis; RF: rectus femoris; VL: vastus lateralis

The shear modulus values of the three muscles are presented in Fig. 2. A significant main effect was noted in terms of group, muscle, and angle; a significant interaction between muscle, angle, and group was also observed (all *p*-values < 0.001). Post hoc analysis revealed that the muscle shear moduli of RF and VL were significantly higher in the senior than the young adults when the knee was positioned at 60°, 90°, and 105° of knee flexion (all *p*-values < 0.006 for RF and < 0.041 for VL). Specifically, at 60°knee flexion, the shear moduli were 34% (*p* = 0.006) and 16% (*p* = 0.031) greater in the senior than the young adults for RF and VL, respectively. At 90° of knee flexion, the shear moduli were 56% (*p* < 0.001) and 13% (*p* = 0.031) greater in the senior adults for RF and VL, respectively. At 105° of knee flexion, the values reached to 68% (*p* < 0.001) and 13% (*p* = 0.041) for RF and VL, respectively. When considering the VM muscle, significant greater shear modulus of the senior adults was only observed at 105° of knee flexion, which was 11% greater than young adults (*p* = 0.020).

**Fig. 3:**
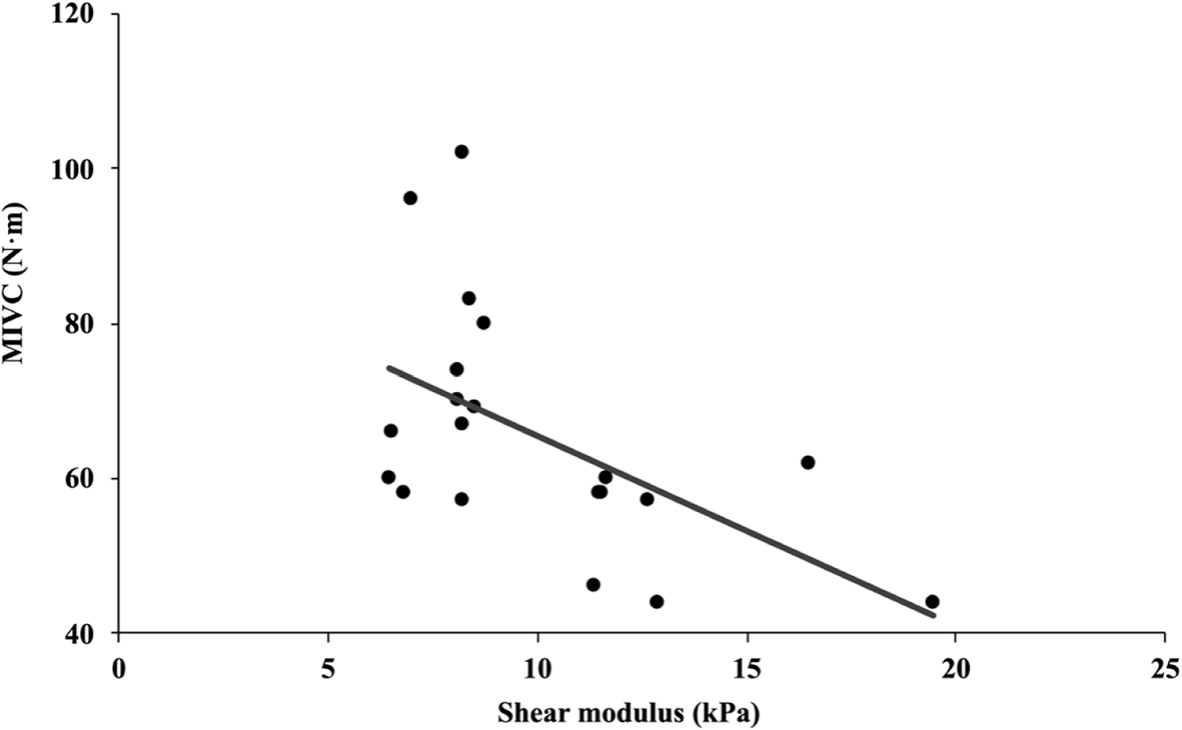
Relationship between passive shear modulus of the rectus femoris muscle and the maximal isometric voluntary contraction torque at 60° of knee flexion in senior adults

The MIVC of the senior adults was significantly lower than young adults at 60° and 90° of knee flexion (Table 2). The correlations between passive shear modulus and MIVC are depicted in Table 3 and Fig. 3. As regards the association between the shear modulus and muscle force, we have only detected moderate and negative association between the passive RF shear modulus at 60° of knee flexion and knee extension force in the senior adults (*r* =  − 0.485, *p* = 0.030). In addition, there was a trend for a significant correlation in the young adults (*r* =  − 0.395, *p* = 0.085). However, no significant association was found for other conditions both in the senior and young adults.

**Table 2:** The MIVC of the young and senior adults (N·m)

Knee angles	Young	Senior	*p*
30°	46.15 ± 9.46	40.40 ± 7.93	0.044
60°	82.40 ± 18.50	65.55 ± 15.52	0.003
90°	95.20 ± 22.50	64.15 ± 16.86	< 0.001

**Table 3:** Correlation coefficients between muscle shear moduli of the superficial heads of the quadriceps and maximal isometric knee extension force (*: *p* < 0.05)

Shear modulus		MIVC of young			MIVC of senior		
		30º	60º	90º	30º	60º	90º
VM	30º	− 0.160			0.163		
	60º		0.350			− 0.091	
	90º			− 0.186			0.024
RF	30º	− 0.052			− 0.008		
	60º		− 0.395			− 0.485*	
	90º			− 0.058			− 0.117
VL	30º	0.157			− 0.166		
	60º		− 0.015			− 0.280	
	90º			0.102			− 0.297

## Discussion

In this study, we aimed to assess age-related changes in muscle stiffness in the female senior adults. We have chosen the quadriceps muscle for analysis because of its importance in daily function. Findings from this study were in accordance with our hypothesis that the senior adults had higher muscle stiffness at long muscle length. More importantly, RF muscle stiffness was negatively associated knee extension force.

In this study, we assessed passive muscle stiffness of the superficial heads of the quadriceps muscle at its shortened to lengthened positions by varying the knee positions. The senior female adults exhibited greater muscle stiffness at the RF and VL muscle heads when the muscles were positioned over 60° of knee flexion (knee extension = 0°) and at the VM muscle at a more lengthened position (105° of knee flexion). This present result is consistent with previous studies reporting higher passive stiffness of aged muscles at long muscle length in either animals [11, 28] or humans [16]. However, the difference was not observed when assessed at shortened muscle length [11, 16, 22]. In this connection, it is well known that change in muscle length affects passive muscle stiffness [26, 29, 30]. The muscle fiber will become taut and even reach to a cross-bridges detached length when the muscle is stretched beyond its slack length which is just over 40° of knee flexion for quadriceps muscle [26, 31].

The underlying mechanisms responsible for the observed increased passive stiffness in the senior female adults could be multifactorial. These include the increased extracellular matrix with aging, which, in turn, increases the muscle stiffness nearly three times in aged quadriceps femoris muscle [32, 33], the decrease of the amount of highly compliant element in the extracellular matrix such as elastin and collagen type III [28], as well as the changes in muscle fiber type and the declined level of estrogen in senior female adults. It has been reported that the area and the percentage of type I muscle fibers increase with age [34]. In response to passive stretch, the type I muscle fibers in isolated VL muscle exhibit greater passive stiffness than type II fibers [35]. The aged muscles with greater amount of type I muscle fibers might display greater passive stiffness than young muscles. Estrogen deficiency might be another factor that reduces passive muscle stiffness in females by means of mediating collagen content within the muscle [36]. Therefore, estrogen deficiency in senior female adults, in particular, after menopause, might contribute to the increase in passive muscle stiffness.

However, age-related increase in passive stiffness of VM was not identically observed as RF and VL over 60° knee flexion. The possible reason could be the imbalanced reduction in muscle mass with aging. It has been reported that the volume of VM was the least affected when it comes to aging among the four heads of quadriceps femoris muscle [37]. The loss of muscle mass with aging would then be replaced by fat and connective tissue, which, in turn, could induce increase in passive muscle stiffness [8]. The relative preservation of VM volume might be one of the reasons for the less increased stiffness at 60° and 90° knee flexion.

We have also detected a negative relationship between passive stiffness of RF and the maximal isometric extension force in senior adults. More specifically, individuals with stiffer RF would produce lower knee extensor force when measured at 60° of knee flexion. In older adults (mean age of 73), no association between the RF stiffness and quadriceps extension strength was observed [15].The authors used strain ratio as an index of passive muscle stiffness. The strain ratio was computed from the strain of the muscle to that of an acoustic coupler. More importantly, the strain of the muscle was measured at its shortened length (knee in extension). Similarly, we could not delineate a significant relationship between muscle stiffness of RF when measured at 30° of flexion and knee extension force.

As muscles with higher echo intensity displayed lower muscle shear modulus and greater muscle strength [38], and echo intensity of RF was negatively associated with maximal isometric force at 60° of knee flexion and functional performance [39, 40], it might be possible that muscle stiffness of RF is associated with knee extension force. However, further investigation is required to understand the underlying mechanism of this correlation.

This study had limitations. First, the results of this present study should be interpreted in regard to the characteristics of our population, i.e., females aged between 50 and 70 years old. This age group was examined because of its association with muscle atrophy that almost apparently occurred in all the skeletal muscles by 50 years old [41]. It is likely that the effect of aging on increased passive muscle stiffness would be more noticeable in more advanced age (> 70) as muscle shear modulus increased with age in person aged more than 60 years old [16], but it remains to be investigated. Second, only females were examined in this study. Thus, in the future, studies examining the age-related alteration on muscle stiffness in males and its effect on muscle force should be conducted. Another limitation is that a rest time of 30 s between passive shear modulus assessments at each angle might be inadequate for stable muscle stiffness after position change due to stress-relaxation effect. In addition, we measured active muscle torque from all the knee extensor groups, but only measuring stiffness from a single/superficial muscle group. Also, the deep quadriceps femoris muscle head (vastus intermedius) was not examined in this study due to its deep position.

## Conclusions

This present study has demonstrated that senior females had greater passive stiffness at the superficial heads of the quadriceps muscle when measured at long muscle length. In the senior female adults, the passive stiffness of RF muscle was found to be negatively associated with the knee extensor force only at 60° knee flexion. No significant association was found for other angles and muscles. These findings highlight how aging affects muscle stiffness of the quadriceps muscle and possible strategies to keep muscle force for senior female adults.

## Data Availability

All data generated or analyzed in this study are included in this published article.

## Abbreviations

MIVC: Maximal isometric voluntary contraction
RF: Rectus femoris
VL: Vastus lateralis
VM: Vastus medialis
